# Research on the impact of digital technology applications on firms' dual innovation in the digital economy context

**DOI:** 10.1038/s41598-024-57183-y

**Published:** 2024-03-17

**Authors:** Qiu Peiyao, Chang Benrui

**Affiliations:** 1 Nanjing Normal University, Business School, Nanjing, 210046 P.R. China; 2 Tianjin University, Economics School, Tianjin, 300072 P.R. China

**Keywords:** Digital technology applications, Firms’ dual innovation, Data-driven dynamic capabilities, Environmental complexity, Impact mechanism, Psychology, Mathematics and computing

## Abstract

In recent years, applications of digital technology have gradually become the primary driving force for enterprise innovation. Although the impact of digital technology applications on enterprise innovation has been recognized by the academic community, existing studies have failed to systematically reveal the mechanism of digital technology applications on firms' dual innovation, overlooking the data-driven dynamic characteristics of innovation development in the digital era, and neglecting external conditions such as environmental complexity. Therefore, based on dynamic capability theory and resource orchestration theory, this study adopts a “technology-economy” research paradigm to explore and analyze the impact of digital technology applications on dual innovation. It also analyzes the mediating role of data-driven dynamic capabilities and the moderating effect of environmental complexity. The article conducts an empirical test using microdata of Chinese Shanghai and Shenzhen A-share listed firms from 2010 to 2021.The research results show that digital technology applications positively promote dual innovation of enterprises and exhibit certain heterogeneity. Data-driven dynamic capabilities play a mediating role in the relationship between digital technology applications and dual innovation. The study also tests the moderating effect of environmental complexity and finds that the higher environmental complexity in which the company operates, the stronger the facilitating effect of digital technology applications.

## Introduction

With the rapid development of the digital economy, emerging digital technologies such as artificial intelligence, cloud computing, blockchain and big data have become new driving forces to promote enterprise transformation, and have also brought new forces and opportunities to innovative activities^1^. The global strategic arrangement and implementation of the digital economy are progressing simultaneously, providing essential support for global economic recovery. In 2021, the estimated scale of the digital economy in 47 countries reached 38.1 trillion US dollars, with a nominal growth of 15.6% year-on-year, accounting for 45.0% of GDP. Currently, a tri-polar pattern of global digital economic development has taken shape among China, the United States, and Europe. In terms of scale, the United States maintains its position as the world's largest digital economy, while China ranks second. It is clear that China places great emphasis on the development of the digital economy and has also achieved certain results. Therefore, researching the issues existing in the development of China's digital economy is of great value and significance. Currently, The Chinese government has urged for the acceleration of digital development, the promotion of deep integration between the digital economy and the real economy, and has set the goal of achieving thorough and rapid integration of digitization and technological innovation^2^. This includes advancing integration of data resources and open sharing, in order to foster new momentum for China’s new economic development. According to the “China Digital Economy Development White Paper (2021),” the scale of China's digital economy reached 39.2 trillion CNY in 2020, accounting for 38.6% of GDP. In this context, digital technologies have gradually become the core driving force for corporate innovation. However, in reality, most Chinese companies are still in the exploratory stage of applying digital technologies. Therefore, how companies can seize new technological opportunities, promote digital technology applications, fully leverage the innovative enabling effects of digital technologies, transform traditional innovation models, and improve corporate innovation performance have become key breakthrough points for China to achieve core technological independence and accelerate the realization of its digital power strategy. This is also an important issue of common concern in international academic research and business practice.

In recent years, driven by the digital economy, research on the relationship between applications of digital technology and enterprise innovation has started to receive academic attention. However, there are fewer related studies. Most of existing studies believed that digital technology applications have a positive impact on enterprise innovation performance. In early research on digital technology applications, scholars mostly used theoretical analyses and case studies^3^. Some scholars have elaborated the significant role of digital technology in enterprise innovation at a theoretical level and have discovered that digital technology can support complex business ecological scenarios through its robust “technology penetration,” which can enhance the innovative performance of the enterprise^4^. Some scholars also conducted multiple case studies on companies like Alibaba and ByteDance and found that applications of digital technology can promote changes in business models, create ecological networks, optimize digital capabilities, and thus improve the innovation performance of enterprise^5^. With the increasing research on the applications of digital technology, most of the recent studies are based on quantitative analysis. For example, scholars like SUK I^6^ and WU F^7^ empirically examined the direct relationship between applications of digital technology and enterprise innovation performance using data from listed companies, and found that applications of digital technology positively promote enterprise innovation^6,7^. They emphasized that applications of digital technology can improve business or change the ecosystem and operational processes, enhancing enterprise innovation performance. It is clear that most current academics believed that applications of digital technology can significantly promote enterprise innovation by taking full advantage of the benefits of instant data sharing, interaction, and programmability^8^.

Therefore, previous studies have provided preliminary empirical evidence and theoretical insights into whether applications of digital technology affect enterprise innovation performance^9^. However, there are still some areas that can be improved. Firstly, several previous studies have not yet explored the impact of digital technology applications on firms’ dual innovation, and there are major differences between the two innovation strategies of exploitative and exploratory innovation. Therefore, it is worthwhile to explore in depth how digital technology applications affect dual innovation and whether there are differences in the impact effects. Secondly, while some studies have explored the impact of digital technology applications on enterprise innovation, they mainly focus on its overall driving effect on enterprise innovation activities, and have not thoroughly examined the mechanism and external conditions of digital technology applications affecting enterprise innovation. These provide possible breakthrough space for the research of this article. Data-driven dynamic capability is emerging concept in the digital era, which may serve as a medium to connect digital technology and internal innovation behavior, and help enterprises to diversify the connection of internal innovative knowledge and external heterogeneous innovative knowledge, thus more effectively absorbing and transforming innovative knowledge to promote dual innovation^10^. Therefore, whether the data-driven dynamic capability can become a mediating mechanism between digital technology application and dual innovation is worth further exploration. Moreover, environmental complexity is a very important external environmental factor. Previous studies have concluded that the booming digital economy has become a disruptive driver of the market, changing the boundaries of products and services and having a profound impact on the market environment in terms of consumer behavior, competitive landscape and business models. And the external environment in which enterprise operate is becoming more complex and enterprise need to innovate adaptively^11^. Therefore, it is also worth exploring whether there is a moderating role of environmental complexity. These gaps provide potential avenues for exploration in this study. Therefore, this study aims to address the following research questions:

1. In the context of the digital economy era, how does digital technology applications affect dual innovation (exploitative innovation and exploratory innovation)? Does it promote or hinder innovation?
2. How does data-driven dynamic capabilities mediate the relationship between digital technology applications and dual innovation of firms?
3. Does the moderating effect of environmental complexity exist?

Based on the above analysis, this paper investigates the impact of digital technology applications on firms’ dual innovation based on dynamic capability theory and resource orchestration theory. This paper believes that digital technology has disruptive properties, and the innovation activities triggered by digital technology applications may provide new impetus for enterprise exploratory innovation; at the same time, digital technology is also considered to have connectivity properties, and digital technology applications can help enterprises to achieve user-centric innovation models through digital platforms and other connectivity methods, thus promoting exploitative innovation. Secondly, this paper extends the relevant research on the impact mechanism of digital technology application on enterprise innovation, and believes that data-driven dynamic capabilities may play an intermediary role. On the one hand, applications of digital technology can help enterprise to form innovative adaptive capacity for digital perception, digital capture, and digital absorption supported by data, thereby improving the data-driven dynamic capabilities of the enterprise^12^. On the other hand, the improvement of data-driven dynamic capabilities can help enterprises reconfigure the digital innovation process through information interaction and data linkage, enhance the heterogeneity and combinatorial nature of knowledge, and promote the dual innovation of enterprise. Finally, this paper adds important external conditions, arguing that environmental complexity plays a moderating role. In summary, the innovation of this study lies in researching the impact of digital technology applications on the dual innovation of enterprises, and incorporating the factors of data-driven dynamic capability and environmental complexity into the theoretical framework, providing theoretical support for enterprises to effectively choose digital paths and promote dual innovation.

This paper’s potential research contributions are reflected in four aspects. Firstly, from the perspective of dynamic capabilities, this paper delves into the impact of digital technology applications on dual innovation of enterprises and analyzes in detail the effects of digital technology applications on exploitative innovation and exploratory innovation. Secondly, by introducing data-driven dynamic capability variables, it dissects their mediating role in the relationship between digital technology applications and dual innovation of enterprises, revealing the “black box” of the impact mechanism of digital technology applications on dual innovation of enterprises. Thirdly, this paper analyzes the moderating effect of environmental complexity, incorporating important external conditions into the study and expanding the research boundaries of the relationship between digital technology applications and dual innovation of enterprises. Lastly, this paper innovatively measures the level of digital technology applications in enterprises from two levels, namely the “fundamental digital technology” and the “integration of digital technology and business operations,” providing a more comprehensive measurement of the level of digital technology applications and serving as a reference for subsequent related research.

## Theoretical background

### Data-driven dynamic capabilities

The resource orchestration theory reveals the “black box” process of creating value from resources to capabilities^13^. Building on the basis of the three-stage static resource orchestration actions of resource combination, resource bundling, and capability leveraging, Sirmon et al.^14^ proposed a dynamic evolution model of stages including inception, growth, maturity, and decline, and discussed the evolutionary motives. The dynamic capability theory suggests that higher dynamic capabilities help organizations effectively perceive, capture, and recombine external resources^14^. In the digital economy era, the status of digital resources is becoming increasingly important. The resource orchestration theory and dynamic capability theory provide a dynamic evolutionary model from digital resources to capabilities, serving as the foundation for the formation of digital capabilities for enterprises.

**Figure 1:**
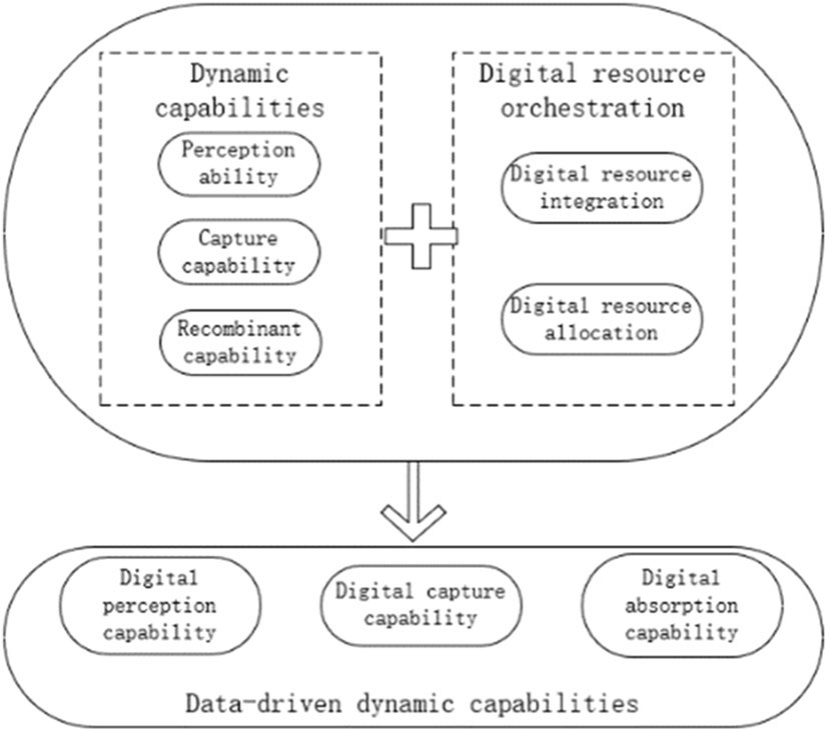
The process of evolution of data-driven dynamic capabilities.

In the era of the digital economy, data-driven dynamic capabilities have emerged as a new concept in the digital age. Warner^12^ and other scholars proposed that data-driven dynamic capabilities involve the reshaping of the perception, capture, and absorption processes of dynamic capabilities by digital resources^12^. Warner also used cross-industry cases to propose the impact of internal and external contingency factors triggering the perception, capture, and absorption processes of digital resources for dynamic capabilities and emphasized the strategic update of data-driven dynamic capabilities. They also provided a model for measuring the evolvement stages. In addition, scholars have researched the composition of data-driven dynamic capabilities. Chirumalla^15^ classified data-driven dynamic capabilities through case studies in process industries, highlighting the relationship with the development of traditional dynamic capabilities^15^. Annarelli^16^ extracted and classified the characteristics of data-driven dynamic capabilities from the concept of data-driven dynamic capabilities through literature analysis^16^. Based on the research by Warner^12^, this paper considers data-driven dynamic capabilities as the reshaping of the perception, capture, and absorption processes of dynamic capabilities by digital resources, specifically divided into digital perception capability, digital capture capability, and digital absorption capability, as shown in Fig. 1. However, existing research has lacked studies on the antecedents and impact effects of data-driven dynamic capabilities, and in particular, whether data-driven dynamic capabilities have an impact on enterprise innovation needs to be further explored.

### Dual innovation

The essence of dual innovation of enterprises is the rational allocation and effective utilization of internal and external resources^17^. These innovations include exploratory innovation and exploitative innovation. Exploratory innovation, also known as breakthrough innovation, refers to the development of completely new products or services based on the reconstruction or even subversion of existing knowledge and technology, in order to meet the needs of new customers and new markets. Exploitative innovation, also known as incremental innovation, refers to the extension or upgrading of the content and improvement of delivery methods of current products or services based on existing knowledge and technology, to further satisfy and expand the existing customer base. How enterprises can mitigate the tension between exploration and exploitation activities and achieve dual innovation has become a core issue in the field of innovation management. Some scholars, based on the resource-based view, have conducted research from the perspective of dynamic capabilities and believe that dynamic capabilities play an important role in dual innovation^18^, helping enterprises integrate and allocate innovation resources more efficiently, there by promoting dual innovation of enterprises. The rapid technological innovation, changes in customer preferences, and shifts in product supply and demand are external dynamic factors that drive dual innovation of enterprises. Actively responding to changes in the market environment to break through the innovation trap is key to maintaining long-term competitive advantage for enterprises.

## Review and theoretical hypotheses

### Digital technology applications and dual innovation

Based on the theories of dynamic capabilities and resource orchestration, this article analyzes the impact of digital technology applications on dual innovation of enterprises from the perspective of dynamic capabilities. In the context of the digital economy, the characteristics of risk ambiguity, dynamic integration, environmental interaction, and time constraints in corporate innovation activities clearly impose higher requirements on dynamic capabilities. Digital technology applications have become the important trigger driving the generation and evolution of dynamic capabilities^12^. Digital technology applications not only help enterprises acquire and integrate internal and external digital resources but also drives them to effectively respond to changes in the external environment, promote the innovation and development of personalized products, and gain innovation advantages^19^. Firstly, through digital technology applications, enterprises creatively form new strategic resource supplies, allowing them to develop a more heterogeneous knowledge base, giving new value and uses to innovation resources, enabling enterprises to identify more heterogeneous opportunities, and alleviating the rigidity of knowledge structures. Furthermore, digital technology applications facilitate the integration and optimization of internal and external innovation resources, leading to significant innovations in research and development strategies, operational processes, and business models, thus laying a solid foundation for enhancing the level of technological innovation and effectively promoting dual innovation of enterprises. It can be seen that in the era of the digital economy, with the continuous maturation of the “technology-economy” research paradigm, digital technology applications drive enterprises to allocate more innovation resources to innovation and development activities, forming a virtuous cycle of internal and external interactions, and promoting dual innovation of enterprises.

Moreover, digital technology applications have influenced both exploratory and exploitative innovation in enterprises. Exploratory innovation emphasizes the exploration of emerging customers and market demands, continuously experimenting with new choices to gain new knowledge, technologies, and skills. Digital technology applications enable enterprises to master unique core technologies and engage in specialized product design, resulting in innovation products with novel and differentiated attributes^20^. Additionally, digital technology applications help enterprises embed technological changes into market-leading products, explore various distribution channels, and develop appropriate and innovative marketing strategies, thereby strengthening the quality of post-sales services for innovative products and effectively communicating with customers to stay abreast of market dynamics. This is crucial for the successful development of innovative products^21^. On the other hand, enterprises adopting an exploitative innovation strategy mainly seek opportunities in their surrounding market environment and retain their basic search activities. Exploitative innovation emphasizes refining and screening existing knowledge and resources, improving existing capabilities, technologies, and paradigms, and acquiring and configuring external resources to unlock their potential value and achieve progressive optimization of products and services. Digital technology applications help enterprises meet the needs of existing customers or markets, enhance product upgrade efficiency, and achieve incremental innovation. Furthermore, digital technology applications can help enterprises reduce the repetition interference of technology and products in product improvement and upgrade by leveraging refined heterogeneous knowledge, thereby improving the efficiency and effectiveness of product upgrades. It is evident that digital technology applications have different effects on exploratory and exploitative innovation in terms of organizational structure, culture, resources, and other aspects, but all have positive promoting effects. Based on the above analysis, this article proposes the following hypothesis:

#### H1a

Digital technology applications positively affect exploitative innovation.

#### H1b

Digital technology applications positively affect exploratory innovation.

### The mediating role of data-driven dynamic capabilities

In recent years, the rapid development of digital technologies represented by artificial intelligence, blockchain, cloud computing, and big data (ABCD) has blurred the boundaries of industries, organizations, departments, and products. These digital technologies have also impacted the capabilities of enterprises^22^. Many scholars believe that digital technology applications can be easily imitated by other enterprises, so it is necessary to transform it into higher-level enterprise capabilities to create value for the company^23^. Data-driven dynamic capabilities are higher-level capabilities that are characterized by their non-imitability, value, scarcity, and non-substitutability. The application of digital technologies in enterprises allows for the integration and configuration of digital resources with internal and external resources that possess characteristics of homogeneity, programmability, and availability, thereby helping enterprises enhance their data-driven dynamic capabilities. Among them, data-driven dynamic capabilities consist of three dimensions of capabilities: digital perception capability, digital capture capability and digital absorption capability.

Firstly, digital technology applications can help enterprises improve their digital perception capability. In the digital economy era, enterprises have effectively improved their real-time interaction and feedback with users through digital platforms and other connection methods. The continuous integration of artificial intelligence, mobile internet, and advanced manufacturing technology also provides enterprises with optimal channel space and information security^24^. In addition, the resource reallocation mode of big data and machine algorithms can drive enterprises to focus on their core business and combine the development of their core business with user feedback, thereby achieving the flexibility in new product research and development. It can be seen that digital technology applications helps enterprises improve their digital perception capability and promotes the implementation of user value-oriented innovation models.

Furthermore, digital technology applications can help enterprises improve their digital capture capability. Unlike traditional innovation, digital technologies leverage their data homogeneity and reprogram ability to provide various channels for enterprises to obtain digital information and resources, thereby enhancing their ability to capture digital opportunities and adapt to market demands and external environment. On one hand, digital technology applications leverage its characteristic of decentralization, using digital platforms to help enterprises achieve freedom in accessing digital and other innovative resources^25^. Through digitizing the value chain and removing intermediaries, enterprises can efficiently utilize their resources and capabilities, thus enhancing their digital resource acquisition capabilities with “architectural advantages”. On the other hand, digital technology applications can reshape the information structure of enterprises and improve the efficiency of information acquisition, promoting real-time sharing and interaction of information. This in turn enhances the integrity and continuity of information acquisition, which is vital for enterprise innovation.

Finally, digital technology applications can help enterprises improve their digital absorption capability. Digital technology applications contribute to enhancing digital absorption capacity in two aspects: organizational management transformation and resource flexibility improvement. From the perspective of organizational management transformation, digital technology applications achieve the interaction of information technology through the integration of digital technologies such as the Internet of Things, big data, and mobile internet. This continuous change in the organizational structure of the company is facilitated by the integration of digital technologies and related technologies. Based on the preset digital management system, enterprises can improve the information interaction capabilities of digital technologies through intelligent and customized information processing^26^. This helps break down the vertical and horizontal boundaries within the company, leading to a flatter and more decentralized organizational structure. This digital organizational management transformation significantly reduces the operational costs of the company, strengthens its data asset management capabilities, and enables precise decision-making in resource allocation, disposal, and retention. It also enhances the connectivity of innovation networks, facilitating a qualitative leap in the company's business models and organizational paradigms, and improving its digital absorption capacity. From the perspective of improving resource flexibility, according to the “environment-strategy-structure” contingency theory, enterprises adjust their strategic direction and organizational models in response to complex external environmental changes. Resource flexibility is about the strategic control and direction of existing resources, and enterprises with high resource flexibility can break free from organizational inertia, leverage high resource conversion efficiency and low resource conversion costs, and achieve digitization of manufacturing processes and services through information sharing and production cooperation mechanisms^27^. Digital technology applications can utilize transactional platforms, innovative platforms, and other digital platforms to integrate and standardize technology innovation, change the form, nature, and structure of resources, and improve the resource flexibility of the company. In particular, digital platforms and technologies will promote the development of enterprise towards a digital ecosystem strategy, accelerate the integration and restructuring of the company's digital resources with other internal and external resources, and improve the company's resource integration capabilities. Furthermore, the virtuality and flexibility of next-generation digital technologies break traditional boundaries of enterprises, improving the speed of resource elements circulation and optimizing resource allocation in the research and production processes. This enhances the company's ability to choose from diverse resources and strengthens the flexibility of its resources.

In conclusion, digital technology applications contribute to the improvement of data-driven dynamic capabilities, including the capabilities of digital perception, digital capture and digital absorption in three dimensions. Based on the analysis above, this article proposes the second hypothesis:

#### H2

Digital technology applications positively affect data-driven dynamic capabilities.

The improvement of data-driven dynamic capabilities also promotes dual innovation of enterprises. The improvement of data-driven dynamic capability helps enterprises efficiently acquire external innovation resources and accelerate the updating of internal knowledge bases, thereby facilitating the collision and fusion of heterogeneous knowledge. This effectively enhances the conversion and absorption of innovative resources, thus promoting dual innovation of enterprises. On one hand, the improvement of date-driven dynamic capabilities promotes efficient external learning and knowledge mining in enterprises^28^. It empowers enterprises to achieve interactive learning between internal cognition and external environment, rapidly connect internal and external digital information sources, and expand the breadth and depth of information. In this process, enterprises continuously absorb, activate, and update innovative knowledge, helping them explore diverse digital resources, accurately acquire and absorb relevant information about customer needs and customer psychology, and actively explore new technological and product knowledge to maximize customer satisfaction and exceed customer experience. Enterprises can also build innovation platforms to incentivize user participation in product design and development, and solicit user creativity. Some scholars have explored the causal relationship between external knowledge acquisition and dual innovation. Some studies have found that external knowledge acquisition has a positive impact on both exploratory and exploitative innovation^29^. Some scholars have also pointed out that external knowledge acquisition has dual characteristics of “exploration” and “exploitation,” which can alleviate and meet the pressures and demands brought by both types of innovation activities, thereby achieving a balance in dual innovation.

On the other hand, the enhanced data-driven dynamic capabilities contribute to the improvement of the conversion and absorption level of innovative resources. For enterprises, the innovative resources obtained from external sources need to be organically embedded and transformed according to the characteristics of the company, and integrated with internal technology in order to be used for innovation within the company. It can strengthen the ability of enterprises to identify, absorb, and utilize external innovative resources. Some academics systematically expounded on the key role of modern digital technology in enhancing the conversion and absorption level of innovative resources for enterprises^30^. If the conversion and absorption level is low, it will be difficult to effectively integrate innovative resources with internal technology, and the effectiveness of business innovation will be greatly reduced. Some studies have combined the conversion and absorption of innovative resources with dual innovation, and some scholars believe that compared to exploitative innovation, conversion and absorption have a greater impact on exploratory innovation. This is because companies that continuously absorb and utilize external innovative resources have a first-mover advantage, can quickly respond to customer needs, and avoid “lock-in effects” and “capability traps”, resulting in innovative products that are significantly different from the current product portfolio and meet new market needs. Some scholars also believe that conversion and absorption have a significant positive impact on dual innovation and can effectively alleviate the tension caused by resource competition in both types of innovation activities^31^. This is because the conversion and absorption of innovative resources not only include imitative learning ability to assimilate and absorb new knowledge from external sources, improve new products or services to meet existing market demands, but also include creative problem-solving ability to create new knowledge that is significantly different from existing knowledge, and develop new products or services to create new market demands. Based on this, the following hypotheses are proposed in this paper.

#### H3a

Data-driven dynamic capabilities positively affect exploitative innovation.

#### H3b

Data-driven dynamic capabilities positively affect exploratory innovation.

To sum up, digital technology applications can help enterprise to improve data-driven dynamic capabilities, including the capabilities of digital perception, digital capture and digital absorption in three dimensions. At the same time, the improvement of digital-driven dynamic capabilities also helps enterprises efficiently acquire external knowledge, and accelerates the updating of their internal knowledge base, promoting the collision and integration of heterogeneous knowledge. This effectively enhances the level of transformation and absorption of enterprise innovation resources, and ultimately promotes dual innovation of enterprises. Based on the analysis, the following assumptions are proposed in this article:

#### H4a

Data-driven dynamic capabilities mediate the relationship between digital technology applications and exploitative innovation.

#### H4b

Data-driven dynamic capabilities mediate the relationship between digital technology applications and exploratory innovation.

### The moderating role of environmental complexity

The concept of environmental complexity refers to the volatility and uncertainty of the external environment in which a company makes decisions. Typically, an increase in the complexity of the external environment can affect the difficulty of formulating and implementing corporate strategies. Therefore, business decision-makers may need to establish alliances or develop common standards to acquire rich information, data, and other resources in order to achieve growth and development^32^. When a company is faced with high levels of environmental complexity, it will encounter strong market competition and urgently need to develop new products to avoid potential losses. In such cases, enterprises need to quickly respond to environmental complexity by leveraging digital technologies. Digital technology applications can help enterprises explore digital resource networks, reshape their organizational models by changing the way they create and capture value, and reduce the “pressure” of coordinating and integrating existing resources in the face of environmental complexity. This, in turn, enhances the flexibility of company resources. Therefore, environmental complexity can be seen as an external factor that motivates enterprises to undergo digital technology applications.

In the face of an increasingly complex and turbulent external environment, competition among enterprises is becoming more intense. Therefore, in order to gain a favorable market position and extend their lifespan, enterprises need to continuously improve their existing technological capabilities and enhance their innovation performance to achieve core competitive advantages. In the era of digital economy, the rapid evolution of digital technology intensifies the instability of the external competitive environment for enterprises^33^. This requires enterprises to have strong internal driving forces to choose the right direction for innovation in platforms and ecosystems, and to shorten the three-stage process of innovation from initiation, development to application as much as possible, in order to enhance the dual innovation efficiency and competitiveness of enterprises. On the other hand, as the level of environmental complexity increases, conducting innovation activities becomes more challenging for enterprises, further motivating them to gain an advantage through digital technology applications. Therefore, the level of environmental complexity strengthens the relationship between digital technology applications and dual innovation of enterprises.

#### H5a

Environmental complexity positively moderates the impact of digital technology applications on exploitative innovation.

#### H5b

Environmental complexity positively moderates the impact of digital technology applications on exploratory innovation.

In the era of the digital economy, applications of digital technology can endow organizations with advanced competitive advantages. The more complex the environment, the more the digital technology empowers the organization's data-driven dynamic capability. The complexity of the external environment is mainly reflected in the high-frequency changes and unpredictability of the environment, which requires companies to react quickly, identify opportunities, and adjust their strategic and innovative models. As a high-level dynamic capability, data-driven dynamic capability aims to explain how enterprises can maintain and create competitive advantages in a dynamically changing environment^34^. Data-driven dynamic capability focuses on the company's ability to adapt to market changes in the face of complex external environments. It is not a static dimension but rather the result of the organization interacting with the external environment. When the environmental complexity is strong, companies are more likely to discover and identify problems through applications of digital technology, make timely adjustments to the organizational structure, adapt to the needs of the external environment, and thus more easily promote data-driven dynamic capability. Simultaneously, the complexity of the environment presents challenges for business innovation, requiring enterprises to seize the opportunity when conducting innovative activities, aligning their own innovative activities with environmental factors^35^. When the environmental complexity is high, environmental factors such as market, technology, and policies do not evolve in a predetermined pattern. Enterprises need to timely and appropriately apply digital technology according to environmental changes to better cultivate data-driven dynamic capabilities. Conversely, when the environmental complexity is weak, as the cultivation of data-driven dynamic capabilities requires enterprises to consume resources such as manpower, materials, and financial resources, enterprises may not prioritize cultivating this capability due to low perceived pressure from the external environment, thus making it difficult to promote data-driven dynamic capabilities. Therefore, environmental complexity also strengthens the relationship between applications of digital technology and data-driven dynamic capabilities.

#### H6

Environmental complexity positively moderates the impact of digital technology applications on data-driven dynamic capabilities.

**Figure 2:**
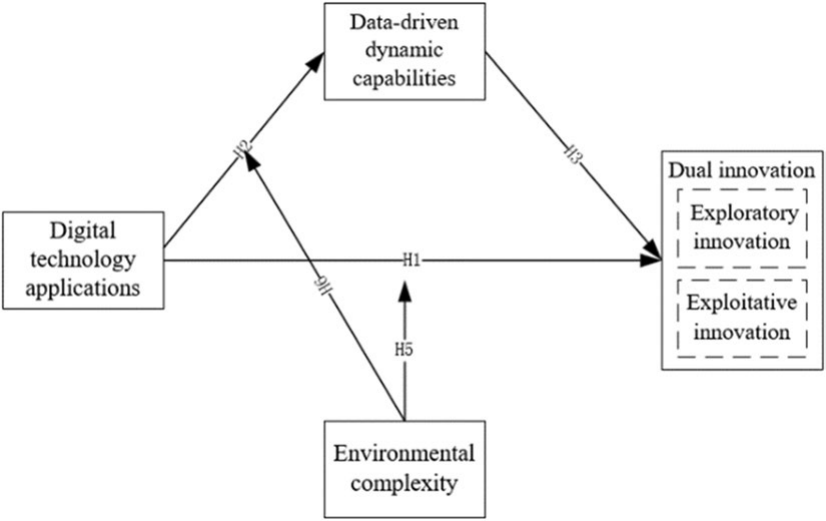
Research framework diagram.

The research framework of this study is shown in Fig. 2.

## Research design

### Sample selection and data sources

In order to test the research hypothesis, this paper adopts a large sample empirical research method. The sample of this study includes some listed companies in China’s A-share market from 2010 to 2021. The following data treatments were applied in this study: (1) Financial industry companies were excluded due to significant differences in capital structure and accounting systems compared to other companies. (2) Companies marked with ST and *ST were excluded from the sample to avoid the influence of companies already in financial crisis or experiencing irreversible deterioration in their operational conditions. (3) Companies with severe missing data on key financial variables were excluded. (4) To mitigate the impact of outliers on the empirical model, all continuous variables were trimmed, with the top and bottom 1% of observations being discarded. The final sample included 1583 companies with 13,709 observations. The sample data were sourced from the CSMAR database, CNRDS database and WIND database.

### Variables definition

#### Explained variables

*Firms’ dual innovation* (*exploratory innovation and exploitative innovation*): Currently, in the academic community, there are two main ways to measure dual innovation, which are the use of financial data and patent data. The former measures exploratory innovation and exploitative innovation through the expenditures on research and development activities, both in terms of expenses and capitalization. The latter measures these types of innovation using different types of patents, with invention patents used to measure exploratory innovation and non-invention patents used to measure exploitative innovation. This study draws on the research by Li and Zheng (2016) and combines it with the characteristics of the existing sample data^17^. Exploratory innovation is measured by the total number of invention patent applications, while exploitative innovation is measured by the total number of utility model patents and design patents. To eliminate the influence of variable absolute scale and avoid negative variable values, this study chooses to take the logarithm of the corresponding patent quantity plus 1 as the final variable value for innovation performance.

#### Explanatory variables

*Digital technology applications* (*DT*): In this study, I referred to the approach of Wu F et al.^7^ to construct a digital technologies applications index for listed companies based on the frequency of keywords related to digital technologies in their annual reports^7^. The first step was to generate a database. I used Python web scraping techniques to collect annual reports of A-share listed companies from 2010 to 2021 on the China Securities Regulatory Commission website. These reports were then converted into txt format using a C ++ program, which served as the database for subsequent keyword selection.

**Figure3:**
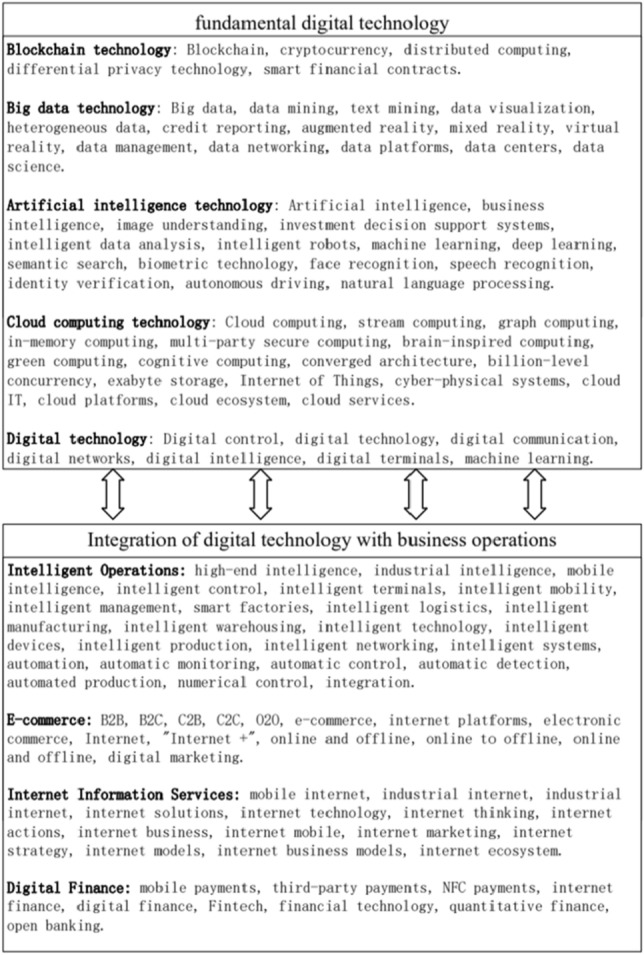
Keyword library for digital technologies applications.

The second step involved determining the keywords. Most of the previous studies select keywords only from the level of fundamental digital technologies. Scholars such as Wu F (2021) and Zhao C^36^ choose Artificial Intelligence, Blockchain, Cloud Computing, Big Data, and other “ABCD “technology-related words as keywords^7,36^. However, there are two shortcomings of previous keyword selection. Firstly, there is no definition of keywords from the two levels of “fundamental digital technologies level” and “integration of digital technologies with business operations level”. Digital technology applications are ultimately all about creating effective innovative outputs and applications in the marketplace, so it is more appropriate to focus on the integration of core digital technologies and front-end business, but the current study lacks the keywords selected from this level. Secondly, the number of keywords is low and does not provide a complete and comprehensive portrayal of the use of digital technologies in business. Therefore, this paper selects key words for digital technology applications from two levels: “fundamental digital technologies level” and “integration of digital technologies with business operations level”. This study supplements and complements the previous keyword library, ultimately forming the keyword library as shown in Fig. 3.

In the third step, the keyword frequency is calculated. Python is used in this study to retrieve, match, and calculate the frequency of keywords, resulting in a cumulative digital technologies applications index. Since the data exhibits a right-skewed distribution, logarithmic transformation is applied in this study.

#### Mediating variables

*Data-driven dynamic capabilities* (*DDC*): Data-driven dynamic capabilities essentially refer to the ability of a company to adapt and evolve over time based on data. Therefore, following the research approach of Lai X^37^, this study aims to measure data-driven dynamic capabilities (*DDC*) using panel data. The data-driven dynamic capabilities (*DCC*) in this paper consists of digital perception capability (*DDC_per*), digital capturing capability (*DDC_cap*), and digital absorbing capability (*DDC_ab*). This paper uses the Delphi method to composite the scores and formulate the weights that can be calculated to obtain the data-driven dynamic capabilities (*DDC*). For the measure of digital perception capability, this study draws on Wang C (2020) research to represent a firm's digital perception capability using the annual number of digital patent citations and applies logarithmic transformation^38^. For the measure of digital capturing capability, this paper uses the proportion of the number of digital technicians to the total number of employees in the company as a measure of digital capturing capability based on Jiang Y^39^. Digital absorbing capability is measured by the intensity of digital R&D expenditure, which is the ratio of annual digital R&D expenditure to operating income.

#### Moderating variable

Environmental complexity (*EC*): Following the research method of Li W^40^, we measure the environmental complexity (*EC*) of a company by using its performance volatility. To ensure more accurate measurement results, we first exclude the stable growth portion of the company's sales revenue and estimate the standard deviation of abnormal sales revenue for the sample companies over the past 5 years. This represents the company's environmental complexity without industry adjustments. Next, we calculate the median environmental complexity of the sample companies in the same industry for each year, which represents the industry’s environmental complexity. Finally, we measure the company's environmental complexity by comparing its environmental complexity without industry adjustments to the industry's environmental complexity.

#### Control variables

Based on relevant literature, this study selects the following control variables: ① Scale: measured by the natural logarithm of total assets at the end of the period; ② Board: measured by the proportion of independent directors to the total number of board members; ③ Top1: measured by the proportion of shares held by the largest shareholder of the company; ④ Leverage: the ratio of total liabilities to total assets; ⑤ Free cashflow: operating cash flow divided by total assets; ⑥ Grow: main business revenue growth rate. The measurement of variables is shown in Table 1.

**Table 1:** Measurement of variables.

Variable type	Variable name	Variable symbols
Dependent variable	Dual innovation (exploratory innovation and exploitative innovation)	IN (exploratory)
		IN (exploitative)
Independent variable	Digital transformation	DT
Mediating variables	Data-driven dynamic capabilities	DDC
Moderating Variable	Environmental complexity	EC
Control Variables	Scale	Scale
	Board	Board
	Top1	Top1
	Leverage	Leverage
	Free cashflow	Free cashflow
	Grow	Grow

### Model design

To test the research hypothesis, this article establishes the following model:

1 $${IN}_{it}={\beta }_{0}+{\beta }_{t}D{T}_{it}+\sum {\beta }_{k}{\text{ Control }}_{it}+\sum \text{ Year }+\sum \text{ Industry }+{\varepsilon }_{it}$$

In the above model, “i” represents the company, “t” represents time. The dependent variable in the regression is dual innovation of the company (*IN*), with digital technologies applications (*DT*) as the core explanatory variable. “Control” is the control variable, and “ε” represents the random error term in the model. To ensure more robust empirical results, the regression model in this study is treated in the following ways: Firstly, to control for the risk of omitted variables, this study simultaneously controls for time fixed effects (*Year*) and industry fixed effects (*Industry*). Secondly, the t-statistics in all regression equations in this study are adjusted using robust standard errors clustered at the company level.

Second, in exploring the mediating effect of knowledge acquisition, this study used a three-step approach to test the mediating effect of knowledge acquisition, as modeled by:

2 $${\text{Mediator}}={\alpha }_{0}+{\alpha }_{1}{\text{ DT }}_{it}+\sum {\alpha }_{k}{\text{ Control }}_{it}+\sum \text{ Year }+\sum \text{ Industry }+{\varepsilon }_{it}$$

3 $${IN}_{it}={\beta }_{0}^{\mathrm{^{\prime}}}+{\beta }_{1}^{\mathrm{^{\prime}}}{DTA}_{it}+{\beta }_{2}^{\mathrm{^{\prime}}}\text{ Mediator }+\sum {\beta }_{k}^{\mathrm{^{\prime}}}{\text{ Control }}_{it}+\sum \text{ Year }+\sum \text{ Industry }+{\varepsilon }_{it}$$

In this case, “Mediator” is the mediating variable of this study, representing data-driven dynamic capabilities (*DDC*). The others variables are consistent with the baseline equation.

Third, the moderating effect of environmental complexity between digital technologies applications and dual innovation was explored. The specific model is as follows:

4 $${IN}_{it}={\alpha }_{3}+{\beta }_{3}{DT}_{it}+\gamma E{C}_{it}+\eta {DT}_{it}\times E{C}_{i,t}+\sum {\beta }_{3k}{\text{ Control }}_{it}+\sum \text{ Year }+\sum \text{ Industry }+{\varepsilon }_{it}$$

“*EC*” represents the complexity of the environment for the businesses, which is the moderating variable in this study. The other variables are consistent with the baseline equation.

Fourthly, the moderating effect of environmental complexity between digital technologies applications and data-driven dynamic capabilities was explored. The specific model is as follows:

5 $${DDC}_{it}={\alpha }_{4}+{\beta }_{4}{DT}_{it}+\gamma E{C}_{it}+\eta {DT}_{it}\times E{C}_{i,t}+\sum {\beta }_{4k}{\text{ Control }}_{it}+\sum \text{ Year }+\sum \text{ Industry }+{\varepsilon }_{it}$$

In this case, “*EC*” represents the complexity of the environment for the businesses, which is the moderating variable in this study. The dependent variable in the regression is data-driven dynamic capabilities (*DDC*). The other variables are consistent with the baseline equation.

## Research results

### Descriptive statistics

Table 2 presents the descriptive statistics for the main variables. Descriptive statistics include the number of observations, mean, standard deviation, minimum value, and maximum value. For the variables of exploratory and exploitative innovation, the mean values are 2.050 and 2.263 respectively, with standard deviations of 1.426 and 1.271. The range of values is from 0 to 5.835 and 0 to 4.571, indicating significant differences in the exploratory and exploitative innovation performance of the sample companies. For the digital technology applications of companies, the mean value is 1.503, with a range of values from 0 to 5.056 and a standard deviation of 1.331. The mean value for data-driven dynamic capabilities is 0.429, with a noticeable difference between the maximum and minimum values of 0 and 0.837, indicating significant developmental differences among different Chinese companies.

**Table 2:** Descriptive statistics.

Variable	Obs	Mean	SD	Min	Max
IN (exploitative)	13,709	2.050	1.426	0	5.835
IN (exploratory)	13,709	2.263	1.271	0	4.571
DT	13,709	1.503	1.331	0	5.056
DDC	13,709	0.429	0.746	0	0.837
EU	13,709	0.035	0.039	0.017	0.081
Scale	13,709	22.020	1.283	19.680	26.030
Board	13,709	0.259	0.438	0	1
Vrd	13,709	35.120	14.960	8.814	74.820
Leverage	13,709	0.427	0.419	0.050	0.894
Free cashflow	13,709	0.084	0.078	− 0.798	0.709
Grow	13,709	0.166	0.101	− 0.618	2.785

### Main effect test

The results of the benchmark regression test are shown in Table 3. Model (1) (2) control for time and industry fixed effects, while Model (3) (4) include additional control variables. The results indicate that the regression coefficients for the level of digital technologies applications are significantly positive at the 1% level, suggesting that an increase in the level of digital technologies applications positively promotes dual innovation in companies. Furthermore, it can be observed that the regression coefficients for the core explanatory variables of exploratory innovation and exploitative innovation are different but both positively significant, providing support for the core hypothesis of this study.

**Table 3:** Main effect test results.

Variables	(1)	(2)	(3)	(4)
	Exploratory	Exploitative	Exploratory)	Exploitative
DT	0.061***	0.049***	0.043***	0.038***
	(0.007)	(0.003)	(0.006)	(0.003)
Scale			0.106*	0.140**
			(0.047)	(0.031)
Board			− 0.008	− 0.006
			(0.041)	(0.063)
Vrd			0.055	0.050
			(0.089)	(0.085)
Leverage			− 0.161**	− 0.127*
			(0.063)	(0.058)
Free cashflow			0.205	0.133
			(0.164)	(0.187)
Grow			0.075**	0.084**
			(0.039)	(0.036)
Year	YES	YES	YES	YES
Industry	YES	YES	YES	YES
_cons	− 0.039**	− 0.083	− 0.075**	− 0.117
	(0.018)	(0.069)	(0.024)	(0.085)
Obs	13,709	13,709	13,709	13,709
${R}^{2}$	0.136	0.194	0.120	0.167

t-statistics in parentheses: * p < 0.1, ** p < 0.05, *** p < 0.01.

### Mediating effect test

The results of the test for mediating effects are shown in Table 4. Model (1), Model (2) and Model (3) show the regression results for exploratory innovation, firstly, Model (1) in Table 4 shows the baseline regression of this paper, which is consistent with the previous results that digital technologies applications significantly promote exploratory innovation. Model (2) in Table 4 indicates a significantly positive regression coefficient, suggesting that digital technologies applications significantly and positively promote firms’ data-driven dynamic capabilities. Model (3) in Table 4 shows the regression of exploratory innovation with digital technologies applications and data-driven dynamic capabilities, with positive coefficients for digital technologies applications and positive coefficients for *DDC*, and both passed the statistical significance test at the 1% level. Compared to model (1), in model (3) with the addition of *DDC*, the coefficient value of digital technologies applications is reduced but still passes the test of significance at the 1% level, indicating that the mediating effect of data-driven dynamic capabilities (*DDC*) exists.

**Table 4:** Mediating effect test results.

Variables	(1)	(2)	(3)	(4)	(5)	(6)
	Exploratory	DDC	Exploratory	Exploitative	DDC	Exploitative
DT	0.037***	0.065***	0.031***	0.040***	0.056***	0.035***
	(0.005)	(0.006)	(0.006)	(0.004)	(0.005)	(0.005)
DDC			0.057***			0.042***
			(0.005)			(0.005)
Controls	YES	YES	YES	YES	YES	YES
Year	YES	YES	YES	YES	YES	YES
Industry	YES	YES	YES	YES	YES	YES
_cons	− 0.063**	− 0.151*	− 0.114	− 0.125	− 0.172	− 0.161*
	(0.027)	(0.059)	(0.136)	(0.072)	(0.094)	(0.056)
Obs	13,709	13,709	13,709	13,709	13,709	13,709
${R}^{2}$	0.139	0.102	0.152	0.196	0.128	0.171

t-statistics in parentheses: *p < 0.1, **p < 0.05, ***p < 0.01.

Model (4), Model (5) & Model (6) are the regression results of exploitative innovation. First, model (4) in Table 4 is the baseline regression of this paper, which is consistent with the previous results. Model (5) in Table 4 indicates a significantly positive regression coefficient, suggesting that digital technologies applications significantly and positively contribute to the data-driven dynamic capabilities of firms. Model (6) in Table 4 shows the regression of exploitative innovation with digital technologies applications and data-driven dynamic capabilities; the coefficients of digital technologies applications are positive, as well as the coefficients of *DDC*, and both pass the test of statistical significance at the 1% level, suggesting that the mediating effect of data-driven dynamic capabilities (DDC) exists. To ensure the robustness of the mediating mechanism, this paper uses the Sobel test to test the above mediating paths and they all pass the test.

### Moderating effect test

The results of the moderating effect test are presented in Table 5. Model (1) tested the moderating effect of environmental complexity on the relationship between digital technologies applications and exploratory innovation in companies. According to the test results, the interaction coefficient between environmental complexity and digital technologies applications is 0.186, which is significant at the 1% confidence level and positive, indicating that the moderating effect of environmental complexity is effective. Model (2) tested the moderating effect of environmental complexity on the relationship between digital technologies applications and exploitative innovation in companies. According to the test results, the interaction coefficient between environmental complexity and digital technologies applications is 0.153, which is significant at the 1% confidence level and positive, indicating that the moderating effect of environmental complexity is effective. Hypothesis 5 is validated. It can be seen that in situations of high environmental complexity, companies are required to continuously absorb and create their own technological resources in response to changing demands, and strengthen their digital management capabilities, thereby creating favorable conditions for promoting intelligent transformation through digital innovation. Therefore, environmental complexity can enhance the impact of digital technologies applications on dual innovation in companies.

**Table 5:** Moderating Effect test results.

Variables	(1)	(2)	(3)
	Exploratory	Exploitative	DDC
DT	0.038***	0.013***	0.027***
	(0.006)	(0.003)	(0.005)
EC	− 0.008	− 0.012	− 0.002
	(0.015)	(0.011)	(0.024)
DT $\times$ EC	0.186***	0.153***	0.129***
	(0.017)	(0.014)	(0.026)
Controls	YES	YES	YES
Year	YES	YES	YES
Industry	YES	YES	YES
_cons	− 0.285**	− 0.334	− 0.315
	(0.082)	(0.197)	(0.164)
Obs	13,709	13,709	13,709
${R}^{2}$	0.175	0.146	0.181

t-statistics in parentheses: *p < 0.1, **p < 0.05, ***p < 0.01.

Model (3) tested the moderating effect of environmental complexity on the relationship between digital technologies applications and data-driven dynamic capabilities. According to the test results, the interaction coefficient between environmental complexity and digital technologies applications is 0.129, which is significant at the 1% confidence level and positive, indicating that the moderating effect of environmental complexity is effective. Therefore, environmental complexity can enhance the impact of digital technologies applications on data-driven dynamic capabilities.

## Heterogeneity analysis

Although the above analysis reveals that digital technologies applications can positively impact company's dual innovation, it is still unclear whether this impact exhibits heterogeneity in different contexts, further exploration is necessary. Existing literature provides valuable insights for the heterogeneity analysis in this paper. Firstly, from the perspective of company nature attributes, there are differences between state-owned enterprises and non-state-owned enterprises in terms of resource base, governance structure, market competition environment, and many other aspects^41^. The digitalization motivation of enterprises is influenced by their nature, and compared with state-owned enterprises, non-state-owned enterprises are more motivated to introduce digital technologies. Secondly, from the perspective of high-tech attributes, digital technology applications are the forefront of technological innovation, and high-tech enterprises are more likely to invest corresponding resources to introduce digital technologies and empower innovation activities. Based on the important evidence provided by the relevant literature mentioned above, this paper further examines the influence of company nature attributes, manufacturing industry attributes, and high-tech attributes on the relationship between digital technologies applications and company's dual innovation.

### Property rights

This article distinguishes the samples into state-owned enterprise group and non-state-owned enterprise group based on the nature of the companies. The regression results are shown in Table 6. To compare the differences between the two groups, the coefficient of variation between groups is introduced, and the Bootstrap method is used to test the difference in coefficients between groups. The number of samples is set to 1000 times, and the final difference coefficient between groups is significant at the 1% significance level. By comparing the coefficient values of the two groups, it can be observed that the optimization effect of digital technologies applications is more pronounced in the non-state-owned enterprise group. This may be because the market competition among non-state-owned enterprises is usually more intense. To avoid being eliminated from the market due to insufficient innovation capabilities, non-state-owned enterprises are often more sensitive and attentive to changes in the external market environment and technological environment, and they need to utilize digital technologies to promote research and development innovation activities.

**Table 6:** Test results based on different property rights.

Variables	State-owned enterprise		Non-state-owned enterprise	
	Exploratory	Exploitative	Exploratory	Exploitative
DT	0.026***	0.014***	0.047**	0.021***
	(0.006)	(0.004)	(0.005)	(0.004)
Controls	YES	YES	YES	YES
Year	YES	YES	YES	YES
Industry	YES	YES	YES	YES
_cons	− 0.453*	− 0.285	− 0.281	− 0.337**
	(0.176)	(0.183)	(0.179)	(0.143)
Obs	4729	5037	6984	7871
${R}^{2}$	0.186	0.273	0.232	0.260

t-statistics in parentheses: *p < 0.1, **p < 0.05, ***p < 0.01.

### High-tech characteristics

The study divided the samples into two groups, based on whether the company is a high-tech enterprise or not. Regression analysis was performed separately for each group, and the results are shown in Table 7. To compare the differences between the two groups, the coefficient of variation was introduced and a bootstrap method was used to test the differences in the coefficients between groups. The number of samples was set at 1000, and the coefficient differences between groups were found to be significant at a 1% level of significance. By comparing the coefficient values between the two groups, it can be observed that the driving effect of digital technologies applications is more pronounced in the group of high-tech enterprises, regardless of whether it is exploratory innovation or exploitative innovation. This may be due to the strategic orientation and production management strategies of high-tech enterprises, which focus on technological innovation and have a high level of innovation activity. These enterprises attach great importance to technological innovation and have strong overall innovation capabilities. They are willing to invest a lot of resources in digital technologies applications and view it as a common understanding among high-tech companies.

**Table 7:** Test results based on different high-tech characteristic.

Variables	High-tech enterprise		Non- high-tech enterprise	
	Exploratory	Exploitative	Exploratory	Exploitative
DT	0.038***	0.017***	0.024**	0.013***
	(0.005)	(0.003)	(0.006)	(0.003)
Controls	YES	YES	YES	YES
Year	YES	YES	YES	YES
Industry	YES	YES	YES	YES
_cons	− 0.219	− 0.185	− 0.252*	− 0.162
	(0.168)	(0.124)	(0.117)	(0.136)
Obs	4905	5608	6817	7327
${R}^{2}$	0.149	0.196	0.153	0.221

t-statistics in parentheses: *p < 0.1, **p < 0.05, ***p < 0.01.

## Robustness test

### Alternative the core explanatory variable

The entropy weight method is used to measure the digital technologies applications index, where the keyword frequency of enterprise digital technologies applications is used as the core explanatory variable in the baseline regression. However, different types of keywords have different impacts on digital technologies applications. Therefore, in the robustness test, the entropy weight method is used to re-measure the variable of enterprise digital technologies applications. The entropy weight method is an objective weighting method. When using the entropy weight method to measure digital technologies applications, the entropy weight of each indicator is calculated based on the frequency of each keyword using information entropy, and then weighted and summed to obtain a new digital technologies applications index (DT1). The regression results, as shown in Table 8 (1) (2), indicate that the digitally transformed measured using the entropy weight method still has a significant positive impact on dual innovation of enterprises. After replacing the core explanatory variables, this paper also tests the mediating and moderating effects, and the test results show that the mediating effect of data-driven dynamic capabilities still exists, and that environmental complexity positively moderates the relationship between digital technology application and dual innovation, and environmental complexity also positively moderates the relationship between digital technology application and data-driven dynamic capabilities. However, due to space limitations this paper will not show the test results one by one, if needed the authors can provide. As can be seen, the conclusions of this paper remain robust.

**Table 8:** Robustness test results.

Variables	(1)	(2)	(3)	(4)
	Exploratory	Exploitative	Exploratory	Exploitative
DT			0.021**	0.016***
			(0.005)	(0.004)
DT1	0.063**	0.045*		
	(0.027)	(0.018)		
Controls	YES	YES	YES	YES
Year	YES	YES	NO	NO
Industry	YES	YES	NO	NO
Year × Industry	NO	NO	YES	YES
_cons	− 0.219	− 0.185	− 0.252*	− 0.162
	(0.168)	(0.124)	(0.117)	(0.136)
Obs	13,709	13,709	13,709	13,709
${R}^{2}$	0.149	0.196	0.153	0.221

t-statistics in parentheses: * p < 0.1, ** p < 0.05, *** p < 0.01.

### Replacement of regression models

The robustness of the model was tested by replacing it with a higher-order joint fixed effects model. The model was examined using the higher-order joint fixed effects method, controlling for “Year × Industry”. The regression results, as shown in Table 8 (3) (4), support the robustness of the conclusions made in this paper. This research not only tested the main effect, but also tested the mediating effect and moderating effect, the test results have verified the hypotheses in the previous paper. However, due to space limitations this paper will not show the test results one by one, if needed the authors can provide. It can be seen that the test results of this paper are still robust.

### Endogeneity test

This article may have endogeneity issues due to sample selection and bidirectional causality. To address this, the instrumental variable method is used for testing and correction. In this paper, we refer to the research methods of Zhao C (2021), and select the number of mobile phone subscribers (Mobile) in cities as an instrumental variable for the 2SLS test^42^. The main reasons for selecting the mobile phone data of each city as an instrumental variable are: firstly, traditional communication technology is related to the application of digital technology, and factors such as the technical level of the local telecommunication infrastructure will affect the application of digital technology; secondly, traditional telecommunication tools, such as the telephone, are not related to the innovation of the enterprise, which ensures the exclusivity of the instrumental variable. Table 9 presents the regression results. Model (1) and model (2) show the results of the first-stage regression, indicating that the instrumental variable Mobile has a significant positive impact on digital technologies applications, confirming the instrument's relevance. Furthermore, the instrumental variable passes the tests for overidentification (Anderson's canonical correlation statistic for exploratory = 126.362, p < 0.01; Anderson's canonical correlation statistic for exploitative = 102.457, p < 0.01) and weak instrument (first-stage F statistic for exploratory = 139.358; F statistic for exploitative = 122.481). Model (3) and model (4) present the estimation results for the second stage, showing that the coefficients for digital technologies applications are all significantly positive, consistent with the empirical results mentioned earlier.

**Table 9:** Endogeneity test results.

Variables	(1)	(2)	(3)	(4)
	Exploratory	Exploitative	Exploratory	Exploitative
DT			0.064**	0.051***
			(0.022)	(0.028)
Mobile	0.274***	0.291***		
	(0.062)	(0.057)		
Controls	YES	YES	YES	YES
Year	YES	YES	YES	YES
Industry	YES	YES	YES	YES
_cons	− 0.142	− 0.189	− 0.117	-0.273
	(0.138)	(0.174)	(0.185)	(0.127)
Obs	10,273	10,882	10,273	10,882
${R}^{2}$	0.153	0.182	0.128	0.246

t-statistics in parentheses: * p < 0.1, ** p < 0.05, *** p < 0.01.

## Discussion

How digital technology applications affect enterprise innovation has been a key issue in academic research in recent years, and scholars have made various studies on this issue. In the early research on the applications of digital technology, scholars mostly used theoretical analysis and case studies. As the applications of digital technology continue to penetrate into all aspects of the enterprise, scholars have gradually deepened their research on digitalization, and in recent years, most of the research has been based on quantitative analysis. Existing research, both theoretically and practically, has provided reference for the exploration of the relationship between digital technology applications and related variables. And regarding the research on the key issue of how digital technology applications affect enterprise innovation, most scholars focused their attention on the use of digital technology for technological innovation, organizational change, business model innovation and synergistic development, and believed that the applications of digital technology can promote enterprise innovation to a certain extent^43,44^. However, there are fewer studies on the impact of digital technology applications on dual innovation of enterprises, and the studies on its mediating mechanism lack the exploration of the intrinsic capabilities of enterprises. Meanwhile, enterprises are placed in the external environment, which is certainly affected by the complexity of the environment, but few scholars have paid attention to the impact generated by external conditions.

Based on this, the research of this paper well improves the research on the relationship between digital technology applications and firms' dual innovation. Firstly, based on the dynamic capability theory and the resource orchestration theory, this paper examines the impact effect of digital technology applications on firms' dual innovation (exploratory innovation and exploitative innovation), and finds that the digital technology applications promote dual innovation. Secondly, this paper enriches and expands the research on the impact mechanism of digital technology applications on firms’ dual innovation by adding the intrinsic capability factor of data-driven dynamic capability. Thirdly, this paper adds environmental complexity as an external condition, and argues that environmental complexity can positively adjust the relationship between digital technology applications and firms' dual innovation, as well as positively adjust the relationship between digital technology applications and data-driven dynamic capabilities. In this study, the internal capability factor of data-driven dynamic capability and the external condition of environmental complexity are incorporated into the theoretical model of the relationship between digital technology applications and firms' dual innovation, so as to further expand the relevant theoretical perspective.

## Conclusions and recommendations

Digital technology applications have significant implications for helping enterprises gain sustainable competitive advantages, driving technological advancements in industries, and even improving national competitiveness. This study examines the impact of digital technology applications on firms’ dual innovation, as well as the mediating mechanism of data-driven dynamic capabilities and moderating effect of environmental complexity, based on the data of A-share listed companies in the Shanghai and Shenzhen stock markets from 2010 to 2021. The research findings are as follows: (1) Digital technology applications significantly promote firms’ dual innovation, and this effect shows heterogeneity, with non-state-owned enterprises and high-tech enterprises being more able to promote firms’ dual innovation through digital technology applications. Enterprises can continuously analyze changes in the internal and external environments through the applications of digital technology, quickly obtain innovation opportunities that are beneficial to their own development, and endow innovation resources with new values and uses, thus helping them to improve their dual innovation. This finding enriches and extends research on the effects of digital technology applications on firms' dual innovation. (2) Digital technology applications can promote firms’ dual innovation by enhancing enterprise data-driven dynamic capabilities. Previous studies have lacked to explore the mediating role of data-driven dynamic capabilities in the relationship between digital technology applications and firms' dual innovation, which also provides space for this paper to make a possible breakthrough. This paper argues that data-driven dynamic capabilities, as an emerging concept in the digital era, can help firms establish diverse connections between internal innovation knowledge and external heterogeneous innovation knowledge, as well as absorb and transform them more efficiently, thus playing a partly mediating role between digital technology applications and firms' dual innovation. (3) Environmental complexity plays a positive moderating role in the relationship between digital technology applications and firms' dual innovation; and positively moderates the relationship between digital technology applications and data-driven dynamic capabilities. The external environment in which an enterprise is located has a certain impact on enterprise innovation. In the face of an increasingly complex and turbulent external environment, the competition among enterprises becomes more intense, and it becomes more challenging for enterprises to carry out innovation activities, which further motivates enterprises to gain innovation advantages through the applications of digital technology. Thus, environmental complexity strengthens the relationship between digital technology applications and firms' dual innovation, as well as the relationship between digital technology applications and data-driven dynamic capabilities.

The contribution of this study is mainly reflected in the following two aspects: firstly, it makes up for the lack of existing studies that pay less attention to the relationship between digital technology applications and firms' dual innovation at the micro level. This study expands the research on the impact mechanism of digital technology applications on firms' dual innovation by adding data-driven dynamic capabilities as an intrinsic capability factor, and it also adds environmental complexity as an important external condition. It can be seen that this study incorporates the data-driven dynamic capability factor and the environmental complexity factor into the theoretical framework of the relationship between digital technology applications and firms' dual innovation, enriching the theoretical explanation between the two relationships. Secondly, the integration of dynamic capability theory, resource orchestration theory and digital technology applications expands the scope of application of traditional theories and injects new perspectives into enterprise digitalization research and enterprise innovation research.

Based on the conclusions of the above research, in order to promote digital technologies applications and upgrade dual innovation of enterprises, this article puts forward the following policy suggestions from the perspectives of enterprises and government: Firstly, accelerate the implementation of the digital development model for enterprises. Enterprises should leverage the comparative advantages of new generation digital technologies in the process of digitization and informatization, create a favorable environment for digital innovation and operation, and further achieve comprehensive interconnection between people, machines, and objects. In the business logic driven by user value, enterprises should explore and innovate in the core areas of digital technologies, pay attention to the cultivation of digital talents, improve their own risk management system, and maximize the goal of dual innovation. Secondly, deepen the digitization and digital industrialization of industries. Regarding industrial digitization, on the premise of ensuring data security and interoperability, the industry should strengthen resource integration and data sharing, and implement big data strategies within the industry. By optimizing and predicting data resources in the aggregation of the information network space, it promotes the upgrading of the digital technology applications of industrial clusters. For digital industrialization, different industries such as IT industry, new energy industry, etc. should actively engage in cross-domain and cross-system digital businesses, build multi-party digital platforms for inter-industry interaction, and enhance the wide-ranging application and deep integration of cross-industry digitalization. Thirdly, create a favorable environment for digital technologies infrastructure. The government should coordinate and promote the external environment, digital technologies applications, and strategic changes in a mutually reinforcing manner. While stabilizing the external market environment, it should also consider enterprise strategic changes and make strategic changes serve the digital economy, providing a fertile ground for the digital economy. It is necessary to quickly adapt to the pace of market digital technologies applications, create a favorable environment for digital development, realize digital participation among different departments, and promote the quality transformation, operational efficiency transformation, and driving capability transformation of the economic and social development.

## Data Availability

The datasets used or analyzed during the current study available from the corresponding author on reasonable request.
